# Management of Aberrant Internal Carotid Artery Injury Caused During Otologic Procedures: Systematic Review and Multicenter Case Series

**DOI:** 10.3390/jcm14155285

**Published:** 2025-07-26

**Authors:** Andreas Spörlein, Susan Arndt, Till F. Jakob, Antje Aschendorff, Theo Demerath, Christian Taschner, Andrzej Balcerowiak, Patrycja Rusin, Ann-Kathrin Rauch, Wojciech Gawęcki

**Affiliations:** 1 Department of Otorhinolaryngology, Medical Center and Faculty of Medicine, University of Freiburg, 79106 Freiburg, Germany; 2 Department of Neuroradiology, Medical Center and Faculty of Medicine, University of Freiburg, 79106 Freiburg, Germany; 3 Department of Otolaryngology and Laryngological Oncology, Poznan University of Medical Sciences, 60-355 Poznań, Poland; 4 Faculty of Medicine, Poznan University of Medical Sciences, 61-701 Poznań, Poland

**Keywords:** aberrant internal carotid artery, arterial bleeding, coiling, interventional radiology, paracentesis, myringotomy, myringoplasty

## Abstract

**Background/Objectives:** An aberrant internal carotid artery (aICA) in the middle ear is a rare vascular anomaly with potentially catastrophic consequences if injured during otologic procedures. Given its rarity, standardized treatment recommendations are lacking. This study aims to present four cases of aICA bleeding, systematically review the literature, and evaluate the outcomes of conservative and interventional management. **Methods:** A retrospective review of four patients treated for intraoperative aICA hemorrhage at two tertiary referral centers was performed. A systematic review was conducted following PRISMA guidelines. Neurologic and otologic outcomes, hemostasis, and complications were analyzed. **Results:** Two patients were treated conservatively with external auditory canal packing, while two required endovascular coil embolization due to pseudoaneurysm formation or persistent bleeding. One patient suffered a stroke due to traumatic ICA occlusion. The systematic review identified 20 additional cases. Conservative treatment alone sufficed in 37.5% of cases, whereas 62.5% required vessel occlusion via coiling, balloon occlusion, or stenting. Neurologic complications occurred in 25% of patients, while otologic outcomes varied widely and were inconsistently reported. **Conclusions:** Initial external auditory canal packing and a CT angiogram should be recommended for all patients. Initial conservative management may be appropriate for cases with early hemostasis if close monitoring is ensured. Endovascular treatment is often necessary, particularly in cases of pseudoaneurysm or rebleeding.

## 1. Introduction

An aberrant internal carotid artery (aICA) coursing through the middle ear represents a rare yet critical and often underdiagnosed congenital vascular anomaly [1,2]. This condition is thought to result from the incomplete regression of embryological vessels, leading to an altered ICA pathway through the temporal bone and middle ear cavity [1,3,4]. Others have related the intratympanic course of the ICA to a segmental agenesis, in which the ascending pharyngeal artery, via its inferior tympanic branch, penetrates through Jacobson’s canal (tympanic canal) into the tympanic cavity, entering the intrapetrous carotid canal to give arterial supply to the ipsilateral hemisphere [5,6]. This may help to understand some of the common associated anomalies such as absence of an extracranial orifice of the intrapetrous carotid canal despite presence of the canal itself, and the presence of a stenosis at the site of entry into the petrous bone. This may also explain the presence of pharyngeal branches and/or an occipital artery arising from this vessel.

Although typically asymptomatic, an aICA in the middle ear can present with conductive hearing loss, pulsatile tinnitus, or otalgia [7,8]. A bluish or reddish mass with or without pulsation may be seen on otoscopy in some cases [9]. Diagnosis often occurs incidentally on imaging. It may be encountered during myringotomy—one of the most commonly performed pediatric surgical procedures, primarily indicated for the management of recurrent otitis media with effusion and chronic eustachian tube dysfunction [10,11,12]. Similarly, an aICA may complicate myringoplasty, the surgical closing of a perforation of the tympanic membrane to restore its anatomical integrity and improve hearing, typically using an autologous graft, such as temporalis fascia or tragal cartilage [13,14,15].

The incidence of aICA is extremely low. Multiple large series of temporal bone computed tomography (CT) scans have been published. One series of 248 CTs reported 13 cases of ICA dehiscence, predominantly in patients with highly pneumatized temporal bones, but no instances of aICA [16]. Similarly, no aICA was found in another series of 186 patients [17], whereas another series of 223 identified one case [18]. Around 70% of patients with aICA are female, with 73% affected on the right side, and 15% of cases present with bilateral aICA [4,9].

Fewer than 40 cases of iatrogenic injury to an aICA have been reported in the literature, mostly single-case reports [3,9,19]. Such intraoperative injuries can result in catastrophic hemorrhage, posing significant challenges in achieving hemostasis and preventing secondary complications such as pseudoaneurysm formation, ischemic stroke, or cranial nerve deficits [19]. Due to the limited literature, no standardized treatment algorithms exist. In this study, four aICA bleeding cases managed at two tertiary referral centers are presented. A conservative approach including packing and an interventional approach using coil embolization are compared qualitatively.

Additionally, a systematic review is conducted, including patients bleeding from an aICA during otologic surgery, by trauma, or spontaneously (population), treated by endovascular intervention or surgery (intervention) or managed conservatively (comparison), considering neurologic deficits, hearing preservation, and other complications (outcome). As the objective is a qualitative comparison between interventional and conservative treatment, studies before the first coil embolization for aICA in 1999 [20] were not included. An overview of earlier cases is provided in a 2017 review [3], although it did not follow the PRISMA guideline [21].

## 2. Materials and Methods

A retrospective chart review of all four consecutive cases from August 2022 to September 2024 with aICA bleeding during otologic surgery at two tertiary ENT centers was conducted. Written consent to participate in the study was obtained from all patients.

A systematic literature review was conducted following the PRISMA guidelines [21]. Eligibility criteria included studies published until April 2025 reporting patients of any age with intraoperative bleeding due to an aICA during otologic surgery. The following electronic databases were searched: PubMed, Embase, Web of Science, Cochrane Library, and Google Scholar, from inception to 21 April 2025. Articles in English, German, French, and Polish were considered. The review was not registered.

The search strategy was developed based on the PICO framework and included both controlled vocabulary and free-text keywords. The complete search strategies for each database are provided in Table S1 in the supplement. Study selection was performed independently by two reviewers in two phases. Discrepancies were resolved by consensus or consultation with a third reviewer. Outcome measures including neurologic and otologic outcome as well as other complications were extracted from the studies. Quality assessment was performed using the Joanna Briggs Institute (JBI) critical appraisal tools specific for case reports and case series [22]. In case of missing data, the authors of the publication were not contacted separately. All included studies were synthesized in the form of a table including age, sex, etiology of aICA bleeding, treatment, neurologic, and otologic outcome.

## 3. Results

### 3.1. Case Series

#### 3.1.1. Case 1

**Figure 1:**
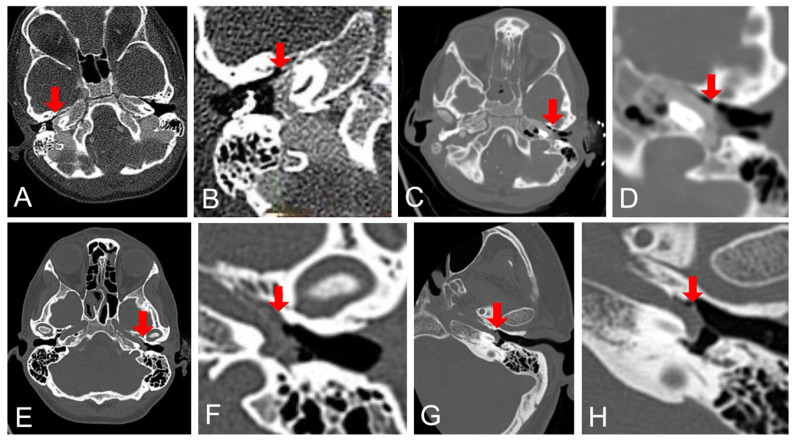
Axial CT temporal bone imaging. Overview and close-up of the tympanic cavity of case 1 (**A**,**B**), case 2 (**C**,**D**), case 3 (**E**,**F**), and case 4 (**G**,**H**), respectively. All aICAs, each with a similar course through the tympanic cavity in a dorsal to anteromedial direction, lateral to the cochlea in front of the promontory, are marked with a red arrow.

A 4.5-year-old boy underwent elective bilateral myringotomy and adenoidectomy for chronic otitis media with effusion and adenoid hypertrophy. Preoperative otoscopy revealed a thickened tympanic membrane on both sides but no other pathological findings. During surgery at an external outpatient facility, profuse acute bleeding occurred from the middle ear. Hemostasis was temporarily achieved with external auditory canal (EAC) packing, and the patient was transferred by air to a tertiary care center while intubated. His hemoglobin level dropped to 8.8 g/dL. Emergency CT angiography identified an aICA coursing through the right middle ear (Figure 1A,B), and he was admitted to the intensive care unit (ICU) for monitoring. Follow-up magnetic resonance (MR) angiography confirmed cessation of bleeding, allowing for the removal of the ear canal packing after two days.

Despite initial hemostasis, re-bleeding occurred following a coughing episode two days after removal of the packing. Angiography identified a post-traumatic pseudoaneurysm of the aICA. Endovascular coil embolization of the right petrous ICA was performed, resulting in complete occlusion. There was robust collateral circulation from the left ICA, and the patient had no neurological deficits after extubation on the following day.

**Figure 2:**
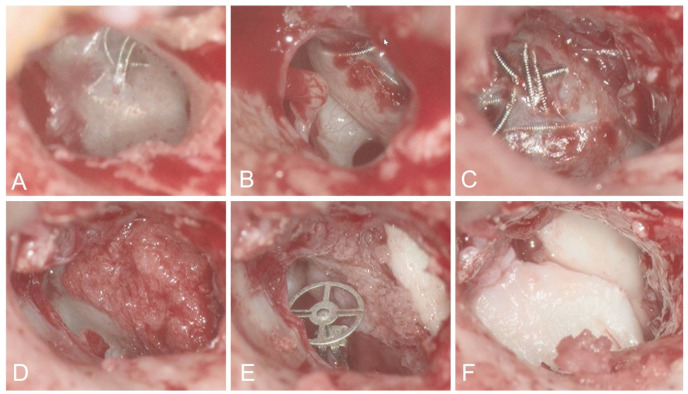
Revision surgery of case 1 due to transtympanic coil migration. Intraoperative microscopy of the intact tympanic membrane with visible extrusion of the coils (**A**) and aICA in the middle ear anterior of the promontory (**B**). Complete exposure of the aICA in the anterior middle ear with exposed coils (**C**). After covering with bone dust in combination with fibrin glue (**D**), placement of partial clip prothesis (**E**), and reconstruction of tympanic membrane with tragal cartilage (**F**).

After six months, otoscopy showed coil extrusion through the tympanic membrane. An MRI ruled out residual flow, and retroauricular tympanoplasty type III was performed with partial ossicular replacement prosthesis (PORP) placement. The exposed coils were covered with the patient’s own bone dust and fibrin glue, as well as tragal cartilage (Figure 2). Audiologic follow-up evaluation revealed no suspicion of sensorineural or conductive hearing loss.

#### 3.1.2. Case 2

A 7-year-old boy with chronic otitis media and conductive hearing loss presented for elective bilateral myringotomy and tympanostomy tube placement due to persistent middle ear effusions and eustachian tube dysfunction. He had previously undergone adenoidectomy and bilateral myringotomy two years ago, as well as repeated adenoidectomy and tympanic tube placement one year ago. Preoperative examination included nasal endoscopy and audiometric evaluation, which confirmed bilateral conductive hearing loss with a 20 dB threshold and flat, type B tympanograms.

**Figure 3:**
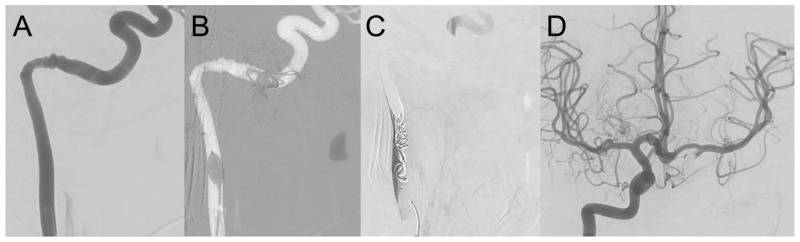
(**A**) Digital subtraction angiography (DSA) of the left ICA of case 2 in a lateral projection showing pseudoaneurysms at the proximal petrous (tympanic) section. (**B**) Coil embolization with bare metal coils was performed from the distal petrous to the distal cervical ICA under proximal flow arrest using a highly compliant dual lumen balloon, since flow-related coil migration to the distal ICA must be avoided at all costs. (**C**) DSA of the left ICA post-embolization in the same projection showing contrast medium stasis proximally, and partial collateralization of the cavernous ICA via external carotid artery branches. (**D**) Substantial collateralization from the right ICA to the left middle cerebral artery via anterior communicating artery is illustrated by DSA with injection from the right ICA.

During myringotomy, profuse arterial bleeding occurred from the left middle ear. Manual compression and EAC packing achieved initial hemostasis. Subsequent CT angiography identified an aICA running through the left middle ear as the source of bleeding (Figure 1C,D). Because of the high risk of re-bleeding and the presence of a large anterior communicating artery segment suggestive of good collateral circulation, a balloon test occlusion was not performed. Immediate coil embolization of the left ICA from the distal petrous to the distal cervical section was conducted via a right femoral access (Figure 3). A highly compliant dual lumen balloon (Scepter CS 4 × 11 mm, Terumo Neuro) was used to achieve flow arrest and to avoid coil migration during the procedure [23]. Post-procedure, he remained stable, with no post-procedural focal neurological deficit after extubation on the same day. On the third postoperative day, the ear packing was removed without complications.

Follow-up six months and one year postoperatively revealed an intact tympanic membrane and an air–bone gap in the lower frequencies up to 1 kHz of 20 dB.

#### 3.1.3. Case 3

A 39-year-old woman was referred to a tertiary ENT center from an outpatient clinic after severe bleeding from the left middle ear during needle puncture of the tympanic membrane for acute otitis media. She complained of pain, feeling of fullness and hearing loss “like underwater” in the left ear for 3 days. Her medical history included lymphocytic leukemia, allogeneic hematopoietic stem cell transplantation, and regular cyclosporine use. At the emergency department, the external dressing was removed to reveal the left EAC filled with blood clots but no ongoing hemorrhage. CT with contrast agent revealed an aICA in the left temporal bone located just below the promontory and minor blood accumulation in the left middle ear (Figure 1E,F). Neurological consultation stated no deficits. The blood hemoglobin was decreased (10.3 g/dL). Due to the lack of active bleeding after admission to the emergency department, the treatment was exclusively conservative and included EAC packing and pharmacotherapy with tranexamic acid. Despite the recommendation for inpatient monitoring, the patient declined admission and was discharged after a 5 h observation. An oral antibiotic with amoxicillin and clavulanic acid, cyclonamin, tranexamic acid, nasal steroid, and constriction drops were recommended. During over two years of follow-up, there was no recurrence of bleeding. The tympanic membrane healed within weeks, although it became noticeably thinner at the puncture site. Pure tone audiometry performed 3 months after the bleeding showed normal hearing in the left ear in the range of 125 Hz to 4 kHz, with a deterioration to 40 dB at 6 kHz and 70 dB at 8 kHz.

#### 3.1.4. Case 4

A 69-year-old woman presented for myringoplasty due to chronic otitis media of the left ear. She had experienced longstanding hearing loss without recent otorrhea or tinnitus. Otoscopic examination showed a large central perforation of the left tympanic membrane mainly in the posterior upper quadrant, with the anterior lower edge adherent to hypertrophic promontory mucosa. Pure tone audiometry confirmed moderate to severe mixed hearing loss in the left ear (air conduction 40–80 dB, air–bone gap 20–40 dB). The scope of the planned procedure did not require preoperative imaging.

Under general and local anesthesia, a skin incision was made in the left EAC. A central perforation of the tympanic membrane was noted, and its edges were refreshed. Upon further dissection of the hypertrophic mucosa from the promontory, a bluish-reddish soft formation resembling a polyp or tumor was revealed. A biopsy attempt led to profuse arterial bleeding, controlled with a hemostatic mesh and an EAC packing with cotton gauze. Myringoplasty was abandoned, and CT imaging was scheduled for the following day.

**Figure 4:**
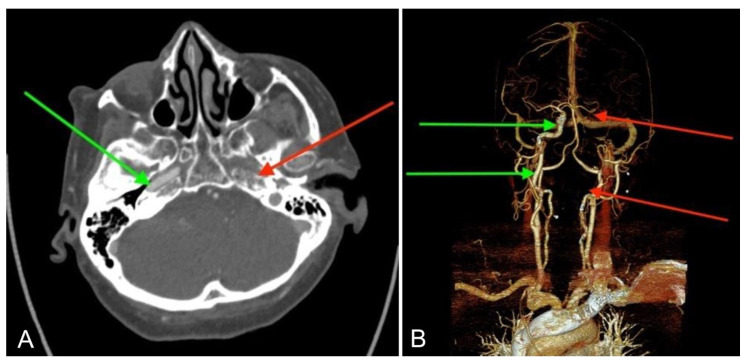
Traumatic occlusion of the left ICA of case 4. CT with contrast 24 h after the middle ear surgery, 1 h after the onset of stroke symptoms. Visible normal, patent right ICA (green arrows) and completely occluded left ICA (red arrows). Axial scan (**A**) and reconstruction of the head and neck arteries (**B**).

On the first day after the procedure, the patient developed confusion and aphasia. Contrast-enhanced CT scans and neurologic consultation confirmed an ischemic stroke in the left cerebral hemisphere with global aphasia and severe right hemiparesis. Angio-CT demonstrated an aICA through the left tympanic cavity in the promontory area with hypoplasia proximal to the tympanic segment, as well as complete occlusion for 25 mm before entering the internal carotid artery canal and in the intracranial section along its entire length (Figure 4). A 12-year-old CT scan, found retrospectively, had also shown the aICA (Figure 1G,H).

Because of the arterial injury and hemorrhage on the previous day, the patient did not qualify for thrombolytic treatment. Pharmacotherapy with enoxaparin, cerebrolysin, infusions of 3% NaCl and ceftriaxone, as well as rehabilitation were implemented. The EAC packing was changed on the fourth day after surgery and then every two days for two weeks. Remnants of the hemostatic mesh were suctioned out four weeks later. During six months of follow-up, no re-bleeding occurred but the patient remained with mild mixed aphasia and right-sided paresis. The tympanic membrane defect has decreased significantly, likely due to the hemostatic mesh. Pure tone audiometry performed 6 months after the bleeding showed severe to profound mixed hearing loss in the left ear (air conduction 50–90 dB, air–bone gap 0–15 dB).

### 3.2. Systematic Review

The initial search yielded 887 records. After removing duplicates, abstract and full text screening, 17 studies met the inclusion criteria (PRISMA flowchart, Figure S1 in the supplement). Exclusion criteria included absence of aICA involvement, lack of reported bleeding, missing treatment details, or publication before the first reported aICA coiling in 1999. Risk of bias was assessed using the JBI critical appraisal tools, with no significant concerns identified; no study was excluded due to high risk of bias (Tables S2 and S3 in the supplement). A total of 24 patients, 20 from the systematic review and 4 from this case series, are synthesized in Table 1. Mean age was 22.9 years, ranging from 1.5 to 69 years. In total, 17 patients (70.8%) were female and only 3 (12.5%) had bilateral aICA. All patients initially received treatment with EAC packing. A conservative treatment was sufficient for nine patients (37.5%), while fifteen patients (62.5%) required some form of vessel obliteration, of which nine (37.5%) were coiled, four (16.7%) underwent balloon occlusion, one (4.1%) was treated with a covered stent, and one required ICA surgical ligation after a failed attempt of a low-flow and high-flow bypass (4.1%). Successful high-flow bypass was achieved in two patients (8.3%). Five patients (25%) suffered neurologic deficits. Otologic outcome is more scarcely discussed. The heterogeneity of patients and treatments does not allow for a quantitative comparison.

## 4. Discussion

The management of iatrogenic injuries to an aICA in the middle ear remains challenging due to the rarity of this anomaly and its potentially catastrophic outcomes. In this study, four cases illustrating different outcomes associated with conservative and interventional approaches are presented, reflecting the variability also observed in the literature. Two of our patients are pediatric, while two are adults. Two cases were treated conservatively, primarily with packing, while two underwent coiling, one primarily and one after failure of packing due to recurrent bleeding. Only case 4 suffered from neurologic impairment, including aphasia and hemiparesis, caused by traumatic occlusion of the aICA.

Conservative management, including EAC packing and close observation, has historically been considered in cases of stable initial hemostasis without significant neurological deficits or evidence of pseudoaneurysm formation [4]. This approach may be preferred if the bleeding is initially controllable, close monitoring is feasible, and the patient’s neurological status remains stable, thereby avoiding the risks of interventional procedures [24]. In case 3, the patient declined further surveillance or intervention, but successful resolution without recurrence of bleeding or neurological complications occurred with EAC packing and pharmacotherapy alone. However, the conservative approach should not be pursued without monitoring and readiness for rapid intervention, as the risk for pseudoaneurysm rupture and subsequent severe hemorrhage or stroke is substantial [3,19]. In multiple cases, a conservative approach had to be abandoned due to recurrent bleeding [20,25,26]. This also occurred in case 1, where conservative measures initially controlled bleeding but failed due to delayed pseudoaneurysm rupture after a coughing episode.

Interventional treatment, including parent vessel occlusion, offers definitive control of hemorrhage and addresses pseudoaneurysms effectively, as corroborated in multiple studies. It can be achieved by either endovascular coiling [1,3,19,20], vascular plug [27], or balloon occlusion [25,28,29,30]. Since detachable balloons are mostly not available anymore [31] and vascular plugs may be difficult to place in tortuous anatomies, platin coils represent the most versatile tool for parent vessel occlusions. Cases 1 and 2 highlight successful hemostasis and immediate resolution of bleeding after coil embolization. Although our two patients treated by coiling had excellent neurologic outcomes, the risk of ischemia in patients with unfavorable collateral circulation remains a relevant issue. Balloon test occlusion of the ICA with or without clinical testing remains controversial in time critical situations. However, it may predict the safety of an ICA sacrifice [32,33]. Coil migration is a rare complication well known from coiling of intracranial aneurysms [33,34], which may lead to infarction of, e.g., the anterior choroidal or the anterior/middle cerebral arteries, with potentially catastrophic outcomes [35,36]. Proximal flow arrest should be used to mitigate this risk [23]. Coil migration through the tympanic membrane was observed in case 1 and had to be treated surgically, combined with tympanoplasty and covering of the exposed coils with stabilizing autologous material.

In select cases, a covered stent may also be used [3,37]. They offer immediate and definitive sealing of the arterial wall. Unlike vessel occlusion methods, covered stents maintain carotid artery patency, thus reducing the risk of cerebral ischemia or stroke, especially when collateral circulation is inadequate. However, the presence of foreign material in the vascular lumen carries a risk of in-stent thrombosis or stenosis, necessitating an effective acute and long-term antiplatelet medication, which might be particularly problematic in pediatric populations [37,38]. Covered stents require larger guiding catheters, which can present significant technical difficulties during navigation and deployment, especially in this application for aICAs [37]. Results on long-term patency, as well as the effect of vascular and skull base growth in pediatric patients on functional outcomes have not been reported [3,19,37].

High-flow bypass grafting from the external carotid artery to the middle cerebral artery with either a radial artery or saphenous vein graft has been used to circumvent the aberrant vessel. Mixed results have been reported, with an excellent outcome in one case [39] and subsequent embolic occlusion leading to limb paralysis in a different case [40].

The ICA can also be ligated or clipped surgically [41,42,43,44,45], traditionally used before the advent of interventional occlusion methods, but can still be used today as an ultima ratio [40]. Depending on the local conditions, an interventional neuroradiologist may not be available [46,47]. Ligation can be combined with otologic surgery including covering of the aICA with bone graft, fascia, muscle, or skin, although the risk for facial nerve injury as well as conductive hearing loss is increased [44,48,49].

Prevention of aICA bleeding remains challenging. Although rare, otologic surgeons must remain vigilant and avoid intraoperative biopsies of red or blue-tinged middle ear masses unless vascularity has been thoroughly assessed. When available, preoperative imaging such as CT or MRI should be carefully reviewed to exclude an aICA, as well as other more common vascular anomalies such as dehiscent high jugular bulb [50,51], glomus tympanicum, or glomus jugulare tumors [52,53,54]. Given the rarity of aICA [16,17,18], routine imaging before procedures like myringotomy is typically not justified due to considerations of radiation exposure, cost, and logistical burden. However, in cases where otoscopy reveals a bluish or reddish mass, especially with pulsations, preoperative imaging should be strongly considered [9]. If an aICA is identified, then surgical intervention should be avoided unless absolutely necessary.

## 5. Conclusions

**Figure 5:**
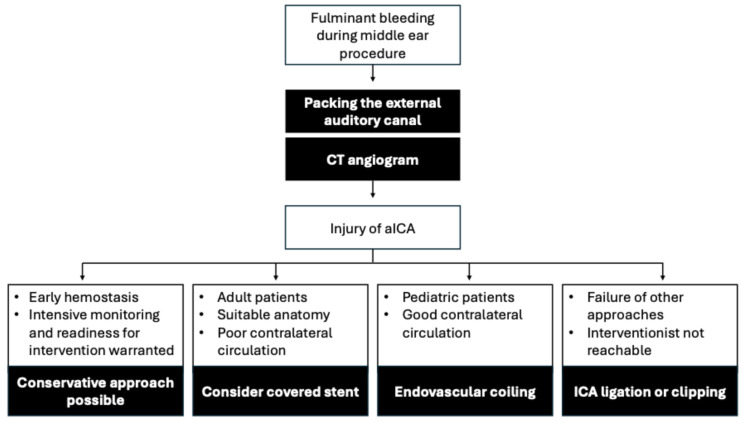
Proposed management approach for aICA causing fulminant bleeding during middle ear procedures.

Packing of the external auditory canal and a CT angiogram is an important first step for all patients with intraoperative bleeding from a suspected aICA (Figure 5). Initial conservative management with packing can be reasonable if immediate hemostasis is achievable, provided that vigilant monitoring, readiness for rapid intervention, and imaging follow-up are guaranteed. However, any suspicion or confirmation of pseudoaneurysm formation, vessel laceration, or unstable clinical course warrants prompt interventional treatment. A covered stent can be considered for select adult patients with suitable anatomy, especially in cases with poor collateral circulation. Parent vessel occlusion by coiling under proximal flow arrest should be preferred for pediatric patients with good collateral circulation. If other measures fail, or if an interventional neuroradiologist is not available, then ICA ligation or clipping should be considered. After intervention, early extubation allows for neurologic assessment. In-patient monitoring for five to seven days should be advised for all patients. Multidisciplinary collaboration between ENT, pediatrics, neuroradiology, and intensive care is crucial for successful management of these cases.

## Figures and Tables

**Table 1:** Table of study characteristics of all studies included in the systematic review with additional information about the four cases of this case series.

Year	First Author	Age	Sex	Etiology	Treatment	Neurologic Outcome	Otologic Outcome
2025	Spörlein (this study)	4	M	Myringotomy	Packing, secondary coiling after recurrent bleeding	No deficit	No hearing loss
		7	M	Repeat Myringotomy	Packing and primary coiling	No deficit	Coil extrusion, air–bone gap in lower frequencies < 20 dB
		39	F	Myringotomy	Packing	No deficit	Normal hearing until 4 kHz, high-frequency hypoacusis
		69	F	Tympanoplasty	Packing	Aphasia, hemiparesis	Deterioration of air conduction by an average of 10 dB and bone conduction by an average of 30 dB, air–bone gap < 15 dB
2022	Wadhavkar	4	F	Myringotomy	Packing and primary coiling	Hemiparesis, gaze deviation	NR
2018	Hudon	7 *	F	Myringotomy	Packing for two weeks	No deficit	Air–bone gap < 40 dB
2017	Kawamura	31	F	Myringotomy	Packing, high-flow bypass with radial artery graft and coiling	No deficit	"Improvement of hearing and resolution of tinnitus"
2017	Bonnard	3	M	Myringotomy	Packing (Planned elective stenting was abandoned after thrombosis and involution of pseudoaneurysm)	No deficit	Air–bone gap 30 dB
2016	Takano	3 *	F	Myringotomy	Packing 1 day, followed by ICA ligation after failed low-flow and high-flow bypass	Hemiparesis	NR
2016	Gnagi	11	M	Repeat Myringotomy	Packing, followed by high-flow bypass	Minor facial nerve weakness	NR
2013	Schutt	3	F	Repeat Myringotomy	Packing 7 days	No deficit	NR
2013	Hirono	54	F	Spontaneous during otitis media	Packing and primary coiling	Slight left hemiparesis	NR
2009	Saylam	28	F	Tympanoplasty	No initial treatment necessary, secondary balloon occlusion	No deficit	NR
2009	Leuin	7	F	Myringotomy	Packing and primary coiling	No deficit	Coil extrusion, mild conductive hearing loss after surgical treatment
2007	Knox	23 *	F	Tympanoplasty	Packing, secondary coiling after recurrent bleeding	No deficit	NR
2006	Sauvaget	37	F	Exploratory tympanotomy	Packing	NR	NR
		34	F	Exploratory tympanotomy	Packing, balloon occlusion	Hemiparesis	NR
		33	M	Exploratory tympanotomy	Packing, coiling	NR	NR
		56	F	Exploratory tympanotomy	Packing	NR	NR
2002	Jain	20	F	Spontaneous, cholesteatoma surgery 3 years before	Packing, primary balloon occlusion	No deficit	NR
2002	Alexander	42	F	Exploratory tympanotomy	Packing, covered stent	No deficit	NR
2000	Hunt and Andrews	1.5	M	Myringotomy	Intraoperative temporary packing	No deficit	No hearing loss
2000	Henriksen	7	F	Repeat Myringotomy	Packing, secondary balloon embolization after recurrent bleeding	No deficit	Temporary 30 dB conductive hearing loss, then normal hearing
1999	Brodish and Woolley	5	M	Myringotomy	Packing, secondary coiling after recurrent bleeding	No deficit	NR

(*) Asterisk behind age means patient hat bilateral aICA. NR: not reported.

## Data Availability

Data from the systematic review are available in the published literature as cited. Additional clinical data from the case series are not publicly available due to patient privacy concerns but may be shared upon reasonable request from the corresponding author.

## Supplementary Materials

The following supporting information can be downloaded at: https://www.mdpi.com/article/10.3390/jcm14155285/s1, Figure S1: PRISMA flowchart for the systematic review; Table S1: Search Strings used for the systematic review in Pubmed, Embase, Web of Science, Cochrane Library, and Google Scholar; Table S2: Joanna Briggs Institute (JBI) critical appraisal checklist for case reports; Table S3: JBI critical appraisal checklist for case series; Table S4: PRISMA checklist.

## Abbreviations

The following abbreviations are used in this manuscript: aICA: aberrant internal carotid artery. CT: computed tomography. CTA: computed tomography angiography. DSA: digital subtraction angiography. EAC: external auditory canal. ENT: ear, nose, and throat. ICA: internal carotid artery. ICU: intensive care unit. JBI: Joanna Briggs Institute. MR: magnetic resonance. MRI: magnetic resonance imaging. PICO: Population, Intervention, Comparison, Outcome. PORP: partial ossicular replacement prosthesis. PRISMA: Preferred Reporting Items for Systematic Reviews and Meta-Analyses.
