# Supplementary material for: Management of Aberrant Internal Carotid Artery Injury Caused During Otologic Procedures: Systematic Review and Multicenter Case Series
Source: J Clin Med. 2025 Jul 26;14(15):5285. doi: 10.3390/jcm14155285 (PMC12347044; doi:10.3390/jcm14155285)
Supplement: Supplementary file 1 [file jcm-14-05285-s001.zip › Table S1 Search Strings.pdf]

| Database | Date | Search String | Hits |
| --- | --- | --- | --- |
| Pubmed | 21.04.25 | ("internal carotid artery"[tiab] OR "carotid artery"[tiab] OR ICA[tiab] OR Carotid Artery, Internal[Mesh])<br>AND (aberrant[tiab] OR ectopic[tiab] OR pseudoaneurysm[tiab] OR Aneurysm, False[Mesh] OR bleeding[tiab] OR aneurysm[tiab] OR Hemorrhage[Mesh] OR injury[tiab])<br>AND (Otologic Surgical Procedures[Mesh] OR myringotomy[tiab] OR myringoplasty[tiab] OR tympanostomy[tiab] OR "middle ear surgery"[tiab] OR "otologic surgery"[tiab] OR "tympanic cavity"[tiab] OR "middle ear"[tiab]) | 172 |
| Embase | 21.04.25 | ( "internal carotid artery".ti,ab. OR "carotid artery".ti,ab. OR ICA.ti,ab. OR exp carotid artery internal/)<br>AND (aberrant.ti,ab. OR ectopic.ti,ab. OR pseudoaneurysm.ti,ab. OR exp false aneurysm/ OR bleeding.ti,ab. OR aneurysm.ti,ab. OR exp hemorrhage/ OR injury.ti,ab.)<br>AND (exp ear surgery/ OR myringotomy.ti,ab. OR myringoplasty.ti,ab. OR tympanostomy.ti,ab. OR "middle ear surgery".ti,ab. OR "otologic surgery".ti,ab. OR "tympanic cavity".ti,ab. OR "middle ear".ti,ab.) | 271 |
| Web of Science | 21.04.25 | TS=("internal carotid artery" OR "carotid artery" OR ICA)<br>AND TS=(aberrant OR ectopic OR pseudoaneurysm OR "false aneurysm" OR bleeding OR aneurysm OR hemorrhage OR injury)<br>AND TS=(myringotomy OR myringoplasty OR tympanostomy OR "middle ear surgery" OR "otologic surgery" OR "tympanic cavity" OR "middle ear") | 144 |
| Cochrane Library | 21.04.25 | #1 internal carotid artery:ti OR internal carotid artery:ab OR carotid artery:ti OR carotid artery:ab OR ICA:ti OR ICA:ab<br>#2 MeSH descriptor: [Carotid Artery, Internal] explode all trees<br>#3 aberrant:ti OR aberrant:ab OR ectopic:ti OR ectopic:ab OR pseudoaneurysm:ti OR pseudoaneurysm:ab OR false aneurysm:ti OR false aneurysm:ab OR bleeding:ti OR bleeding:ab OR aneurysm:ti OR aneurysm:ab OR injury:ti OR injury:ab<br>#4 MeSH descriptor: [Hemorrhage] explode all trees<br>#5 MeSH descriptor: [Aneurysm, False] explode all trees<br>#6 myringotomy:ti OR myringotomy:ab OR myringoplasty:ti OR myringoplasty:ab OR tympanostomy:ti OR tympanostomy:ab OR middle ear surgery:ti OR middle ear surgery:ab OR otologic surgery:ti OR otologic surgery:ab OR tympanic cavity:ti OR tympanic cavity:ab OR middle ear:ti OR middle ear:ab<br>#7 MeSH descriptor: [Otologic Surgical Procedures] explode all trees<br>#8 (#1 OR #2) AND (#3 OR #4 OR #5) AND (#6 OR #7) | 0 |
| Google Scholar (top 300 results) | 21.04.25 | ("internal carotid artery" OR "carotid artery" OR ICA)<br>AND (aberrant OR ectopic OR pseudoaneurysm OR "false aneurysm" OR bleeding OR aneurysm OR hemorrhage OR injury)<br>AND (myringotomy OR myringoplasty OR tympanostomy OR "middle ear surgery" OR "otologic surgery" OR "tympanic cavity" OR "middle ear") | 300 |
