# Supplementary material for: Management of Aberrant Internal Carotid Artery Injury Caused During Otologic Procedures: Systematic Review and Multicenter Case Series
Source: J Clin Med. 2025 Jul 26;14(15):5285. doi: 10.3390/jcm14155285 (PMC12347044; doi:10.3390/jcm14155285)
Supplement: Supplementary file 1 [file jcm-14-05285-s001.zip › Table S2 JBI critical appraisal case reports.pdf]

Year	Author	1.Were patient's demographic characteristics clearly described?	2. Was the patient's history clearly described and presented as a timeline?	3. Was the current clinical condition of the patient on presentation clearly described?	4. Were diagnostic tests or assessment methods and the results clearly described?	5. Was the intervention(s) or treatment procedure(s) clearly described?	6. Was the post-intervention clinical condition clearly described?	7. Were adverse events (harms) or unanticipated events identified and described?	8. Does the case report provide takeaway lessons?	Overall appraisal	Comment
2022	Wadhavkar	Yes	Yes	Yes	Yes	Yes	Yes	Yes	Yes	Include	
2018	Hudon	Yes	Yes	Yes	Yes	Yes	Yes	Yes	Yes	Include	
2017	Kawamura	Yes	Yes	Yes	Yes	Yes	Yes	Yes	Yes	Include	
2017	Bonnard	Yes	Yes	Yes	Yes	Yes	Yes	Yes	Yes	Include	
2016	Takano	Yes	Yes	Yes	Yes	Yes	Yes	Yes	Yes	Include	Otologic outcome not reported
2016	Gnagi	Yes	Yes	Yes	Yes	Yes	Yes	Yes	Yes	Include	Otologic outcome not reported
2013	Schutt	Yes	Yes	Yes	Yes	Yes	Yes	Yes	Yes	Include	Otologic outcome not reported
2013	Hirono	Yes	Yes	Yes	Yes	Yes	Yes	Yes	Yes	Include	Otologic outcome not reported
2009	Saylam	Yes	Yes	Yes	Yes	Yes	Yes	Yes	Yes	Include	Otologic outcome not reported
2009	Leuin	Yes	Yes	Yes	Yes	Yes	Yes	Yes	Yes	Include	
2007	Knox	Yes	Yes	Yes	Yes	Yes	Yes	Yes	Yes	Include	Otologic outcome not reported
2002	Jain	Yes	Yes	Yes	Yes	Yes	Yes	Yes	Yes	Include	Otologic outcome not reported
2002	Alexander	Yes	Yes	Yes	Yes	Yes	Yes	Yes	Yes	Include	Otologic outcome not reported
2000	Hunt and Andrews	Yes	Yes	Yes	Yes	Yes	Yes	Yes	Yes	Include	
2000	Henriksen	Yes	Yes	Yes	Yes	Yes	Yes	Yes	Yes	Include	
1999	Brodish and Woolley	Yes	Yes	Yes	Yes	Yes	Yes	Yes	Yes	Include	Otologic outcome not reported
