# Supplementary material for: Management of Aberrant Internal Carotid Artery Injury Caused During Otologic Procedures: Systematic Review and Multicenter Case Series
Source: J Clin Med. 2025 Jul 26;14(15):5285. doi: 10.3390/jcm14155285 (PMC12347044; doi:10.3390/jcm14155285)
Supplement: Supplementary file 1 [file jcm-14-05285-s001.zip › Table S3 JBI critical appraisal case series.pdf]

Year	Author	1. Were there clear criteria for inclusion in the case series?	2. Was the condition measured in a standard, reliable way for all participants included in the case series?	3. Were valid methods used for identification of the condition for all participants included in the case series?	4. Did the case series have consecutive inclusion of participants?	5. Did the case series have complete inclusion of participants?	6. Was there clear reporting of the demographics of the participants in the study?	7. Was there clear reporting of clinical information of the participants?	8. Were the outcomes or follow up results of cases clearly reported?	9. Was there clear reporting of the presenting site(s)/clinic(s) demographic information?	10. Was statistical analysis appropriate?	Overall appraisal	Comments
2006	Sauvaget	Yes	Yes	Yes	Un-clear	Un-clear	Yes	Yes	Yes	Yes	Not applicable	Include	Otologic outcome not reported, Neurologic outcome explicitly stated only for one patient
