# Supplementary figures and images for: Management of Aberrant Internal Carotid Artery Injury Caused During Otologic Procedures: Systematic Review and Multicenter Case Series
Source: J Clin Med. 2025 Jul 26;14(15):5285. doi: 10.3390/jcm14155285 (PMC12347044; doi:10.3390/jcm14155285)

### Identification of studies via databases and registers

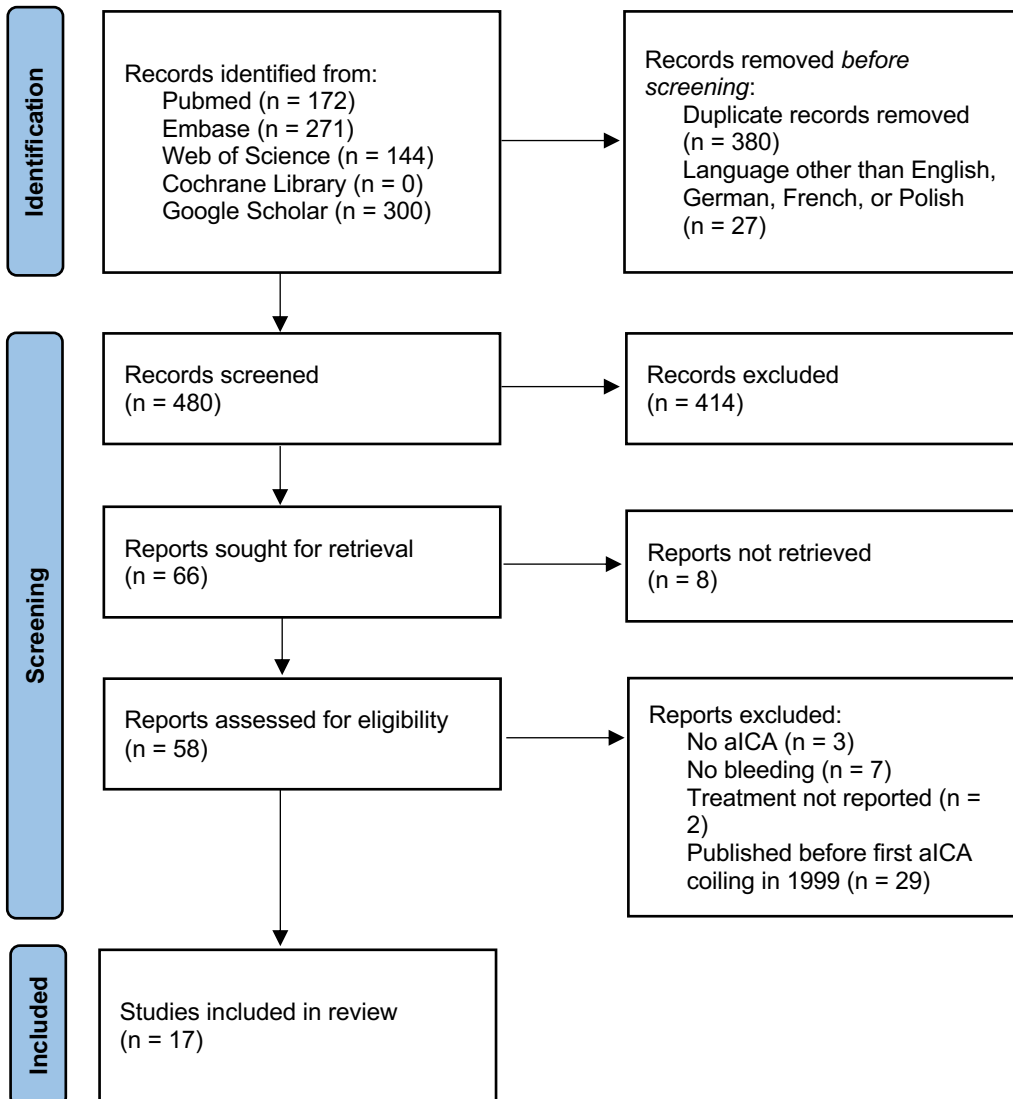

Supplement: Supplementary file 1 [file jcm-14-05285-s001.zip › Figure S1 PRISMA flow diagram.pdf]
